# Performance of chitosan nanoparticle-loaded irrigants on biofilm disruption

**DOI:** 10.6026/973206300213823

**Published:** 2025-10-31

**Authors:** Madhura Kakade, Sonia Sharma, Nagaraja A, Tanushri Pandey, Umesh G, Vinay Rao

**Affiliations:** 1 Department of Conservative Dentistry & Endodontics, CSMSS Dental College & Hospital, Sambhajinagar, Maharashtra, India; 2 Department of Dentistry, Maharaja Agrasen Medical College, Agroha, Haryana, India; 3 Department of Oral & Maxillofacial Pathology, Seema Dental College & Hospital, Rishikesh, Uttarakhand, India; 4 Department of Conservative Dentistry and Endodontics, People's College of Dental Sciences and Research Center, Bhopal, Madhya Pradesh, India; 5 Department of Conservative Dentistry and Endodontics, Bapuji Dental College and Hospital, Davangere, Karnataka, India; 6 Department of Conservative Dentistry, Endodontics and Aesthetic Dentistry, AMC Dental College, Ahmedabad, Gujarat, India

**Keywords:** Chitosan nanoparticles, biofilm disruption, irrigants, antimicrobial, Staphylococcus aureus, Pseudomonas aeruginosa

## Abstract

Biofilm-associated infections pose a major clinical problem due to their resistance to conventional antimicrobial therapies. Therefore, it is of interest to evaluate the effectiveness of chitosan nanoparticle-loaded irrigants in disrupting established Staphylococcus aureus and Pseudomonas aeruginosa biofilms. Using *in vitro* 96-well plate models, biofilm biomass reduction and bacterial viability were assessed with crystal violet assay, CFU counts and SEM imaging after exposure to 2% and 1% chitosan nanoparticles, 0.2% chlorhexidine and saline. Data showed that 2% chitosan nanoparticles produced the greatest biofilm disruption (89.7±3.2%) and bacterial reduction (4.8±0.3 log10 CFU/mL), significantly outperforming 1% chitosan and chlorhexidine. Thus, we show that chitosan nanoparticle irrigants, particularly at higher concentrations, may serve as effective alternatives for managing biofilm-associated infections.

## Background:

Biofilms are structured communities of bacterial cells enclosed in a self-produced polymeric matrix and adherent to an inert or living surface [1]. These microbial communities exhibit remarkable resistance to antimicrobial agents and host immune responses, making biofilm-associated infections particularly challenging to treat [2]. Biofilms are implicated in a wide range of infections, including chronic wounds, dental caries, periodontitis, otitis media and medical device-related infections [3]. The recalcitrance of biofilms to conventional antimicrobial therapies has necessitated the development of novel strategies for their effective disruption and eradication [4]. Chitosan, a natural linear polysaccharide composed of randomly distributed β-(1→4)-linked D-glucosamine and N-acetyl-D-glucosamine, has garnered significant attention in biomedical applications due to its biocompatibility, biodegradability and inherent antimicrobial properties [5]. When processed into nanoparticles, chitosan exhibits enhanced antimicrobial activity attributed to its high surface area-to-volume ratio and improved interaction with microbial membranes [6]. The positive charge of chitosan nanoparticles facilitates electrostatic interactions with negatively charged bacterial cell walls and biofilm matrices, leading to disruption of microbial membranes and leakage of intracellular components [7]. Recent studies have demonstrated the efficacy of chitosan nanoparticles against various microorganisms, including both Gram-positive and Gram-negative bacteria, as well as fungi [8]. In particular, chitosan nanoparticles have shown promise in disrupting biofilms formed by common pathogens such as Staphylococcus aureus, Pseudomonas aeruginosa and Escherichia coli [9]. The mechanisms underlying this activity include penetration of the biofilm extracellular polymeric substance, disruption of quorum sensing and interference with bacterial adhesion to surfaces [10]. Therefore, it is of interest to evaluate the performance of chitosan nanoparticle-loaded irrigants at different concentrations on the disruption of established biofilms, compared to conventional irrigants.

## Materials and Methods:

## Study design:

This was an *in vitro* laboratory study designed to evaluate the efficacy of chitosan nanoparticle-loaded irrigants on biofilm disruption. The study employed a randomized controlled design with four experimental groups and three assessment time points.

## Sample size:

Based on a power analysis with α=0.05 and power=0.90, a sample size of 12 wells per group per time point was determined to detect a 25% difference in biofilm disruption between groups, accounting for potential attrition. The total sample size consisted of 144 wells (4 groups x 3 time points x 12 replicates).

## Inclusion criteria:

[1] Biofilms of Staphylococcus aureus (ATCC 25923) and Pseudomonas aeruginosa (ATCC 27853) aged 48 hours

[2] Biofilms with optical density at 570 nm (OD570) of 0.5 or higher as measured by crystal violet assay

[3] Biofilms formed in 96-well flat-bottom polystyrene tissue culture plates

## Exclusion criteria:

[1] Contaminated biofilms (presence of microorganisms other than the intended species)

[2] Biofilms with inconsistent growth (OD570 < 0.5)

[3] Wells with visible damage or irregularities

## Biofilm formation:

Staphylococcus aureus and Pseudomonas aeruginosa were cultured in TSB at 37°C for 18 hours. The bacterial suspensions were adjusted to 1x10^7 CFU/mL in fresh TSB. 200 µL of each bacterial suspension was added to individual wells of 96-well plates. The plates were incubated at 37°C for 48 hours under static conditions to allow biofilm formation. The medium was replaced every 24 hours to ensure nutrient supply.

## Irrigant preparation:

Chitosan nanoparticles were synthesized using the ionic gelation method. Briefly, chitosan was dissolved in acetic acid solution (1% v/v) to obtain a 0.5% (w/v) solution. Sodium tripolyphosphate (TPP) solution (0.1% w/v) was added dropwise to the chitosan solution under constant stirring at room temperature. The resulting nanoparticles were collected by centrifugation at 15,000 rpm for 30 minutes, washed with distilled water and lyophilized. The nanoparticles were characterized for size, zeta potential and morphology using dynamic light scattering and transmission electron microscopy. For the study, the nanoparticles were suspended in distilled water to obtain 1% and 2% (w/v) solutions.

## Experimental Groups:

[1] Group 1: 2% chitosan nanoparticle-loaded irrigant

[2] Group 2: 1% chitosan nanoparticle-loaded irrigant

[3] Group 3: 0.2% chlorhexidine (positive control)

[4] Group 4: Sterile saline (negative control)

## Biofilm disruption protocol:

After 48 hours of biofilm formation, the medium was aspirated and the wells were gently washed twice with PBS to remove non-adherent cells. 200 µL of the respective irrigant solution was added to each well. The plates were incubated at room temperature for 1, 5, or 10 minutes, after which the irrigant was aspirated. The wells were gently washed twice with PBS to remove any residual irrigant and disrupted biofilm.

## Assessment of biofilm disruption:

## Crystal violet assay:

The remaining biofilm was fixed with 99% methanol for 15 minutes, stained with 0.1% crystal violet for 20 minutes and washed with distilled water. The bound dye was solubilized with 33% acetic acid and the absorbance was measured at 570 nm using a microplate reader. The percentage of biofilm disruption was calculated as: [(OD control - OD test) / OD control] x 100.

## Colony forming unit (CFU) count:

After exposure to irrigants and washing with PBS, 200 µL of PBS was added to each well and the biofilm was scraped and sonicated for 5 minutes to dislodge the bacteria. Serial dilutions were plated on tryptic soy agar and the plates were incubated at 37°C for 24 hours. The number of CFU/mL was determined and the log reduction was calculated.

## Scanning electron microscopy (SEM):

Additional biofilm samples were prepared on sterile glass coverslips placed in 24-well plates. After exposure to irrigants, the biofilms were fixed with 2.5% glutaraldehyde for 2 hours, dehydrated in a graded ethanol series, critical point dried and sputter-coated with gold. The samples were observed under SEM to evaluate structural changes in the biofilm architecture.

## Statistical analysis:

Data were analyzed using SPSS version 25.0 (IBM Corp., Armonk, NY, USA). Normality of data distribution was assessed using the Shapiro-Wilk test. One-way analysis of variance (ANOVA) followed by Tukey's post-hoc test was used for multiple group comparisons. Repeated measures ANOVA were used to compare the effects at different time points. A p-value of <0.05 was considered statistically significant. All data were expressed as mean ± standard deviation (SD).

## Results:

The 2% chitosan nanoparticle-loaded irrigant demonstrated the highest reduction in biofilm biomass across all time points. After 1 minute of exposure, the 2% chitosan nanoparticle group showed 72.4±3.8% reduction in biofilm biomass, this increased to 89.7±3.2% after 10 minutes. The 1% chitosan nanoparticle group exhibited 52.6±4.2% reduction at 1 minute, increasing to 68.4±4.1% at 10 minutes. The chlorhexidine group showed 58.3±3.9% reduction at 1 minute, reaching 72.3±3.8% at 10 minutes. The saline control group demonstrated minimal biofilm disruption, with only 8.2±2.1% reduction at 10 minutes. The differences between the 2% chitosan nanoparticle group and all other groups were statistically significant at all-time points (p<0.001). Viable similar trends were observed in the reduction of viable bacterial counts. The 2% chitosan nanoparticle irrigant achieved a 3.2±0.3 log10 CFU/mL reduction at 1 minute, increasing to 4.8±0.3 log10 CFU/mL at 10 minutes. The 1% chitosan nanoparticle group showed 2.1±0.4 log10 CFU/mL reduction at 1 minute, reaching 3.2±0.4 log10 CFU/mL at 10 minutes. The chlorhexidine group demonstrated 2.4±0.3 log10 CFU/mL reduction at 1 minute and 3.6±0.3 log10 CFU/mL reduction at 10 minutes. The saline control group showed only 0.3±0.1 log10 CFU/mL reduction at 10 minutes. The differences between the 2% chitosan nanoparticle group and all other groups were statistically significant at all-time points (p<0.001). Morphological Changes in Biofilm Structure: SEM analysis revealed significant structural damage to biofilms exposed to chitosan nanoparticle-loaded irrigants. Biofilms treated with 2% chitosan nanoparticles showed extensive disruption of the extracellular polymeric substance, with few intact bacterial cells remaining after 10 minutes of exposure. In contrast, biofilms treated with 1% chitosan nanoparticles and chlorhexidine showed partial disruption, with some areas of intact biofilm architecture. The saline control group exhibited no significant changes in biofilm structure (Table 1-3 - see PDF). All active irrigants demonstrated time-dependent increases in biofilm disruption efficacy. The 2% chitosan nanoparticle group showed the most pronounced time-dependent effect, with a 23.7% increase in biofilm biomass reduction between 1 and 10 minutes of exposure, compared to 15.8% for the 1% chitosan nanoparticle group and 14.0% for the chlorhexidine group. The differences in time-dependent effects between the 2% chitosan nanoparticle group and other groups were statistically significant (p<0.01). The efficacy of the irrigants varied between the two bacterial species tested. Against Staphylococcus aureus biofilms, the 2% chitosan nanoparticle irrigant achieved 91.2±3.1% reduction in biofilm biomass at 10 minutes, compared to 88.3±3.4% against Pseudomonas aeruginosa biofilms. Similarly, the log reduction in viable bacterial counts was 5.0±0.3 log10 CFU/mL for Staphylococcus aureus and 4.6±0.3 log10 CFU/mL for Pseudomonas aeruginosa. The differences in efficacy between the two species were statistically significant (p<0.05).

## Discussion:

The results of this study demonstrate that chitosan nanoparticle-loaded irrigants, particularly at a 2% concentration, exhibit superior biofilm disruption capabilities compared to conventional irrigants. The 2% chitosan nanoparticle irrigant achieved significantly higher reductions in biofilm biomass and viable bacterial counts than the 1% chitosan nanoparticle solution, 0.2% chlorhexidine and saline control at all-time points evaluated. These findings are consistent with previous research highlighting the enhanced antimicrobial activity of chitosan nanoparticles against biofilm-forming microorganisms [11, 12, 13, 14, 15- 16]. The superior performance of the 2% chitosan nanoparticle irrigant can be attributed to several factors. First, the higher concentration provides a greater number of nanoparticles per unit volume, increasing the probability of interaction with the biofilm structure [17]. Second, chitosan nanoparticles possess a positive surface charge that facilitates electrostatic interactions with the negatively charged components of bacterial cell walls and the extracellular polymeric substance of biofilms [18]. This interaction leads to disruption of the biofilm matrix and damage to bacterial cell membranes, resulting in leakage of intracellular components and ultimately cell death [19]. Third, the small size of the nanoparticles (50-100 nm) enables deeper penetration into the biofilm structure, reaching bacteria that might be protected from conventional antimicrobial agents [20]. The time-dependent increase in biofilm disruption efficacy observed with all active irrigants is consistent with the mechanism of action of these agents. Biofilm disruption is a complex process that involves penetration of the extracellular polymeric substance, interaction with bacterial cells and disruption of the biofilm architecture [21]. The 2% chitosan nanoparticle group showed the most pronounced time-dependent effect, suggesting that the nanoparticles continue to interact with and disrupt the biofilm over time, rather than acting immediately upon contact [22]. This sustained activity may be advantageous in clinical settings where prolonged exposure to the irrigant is possible. The species-specific differences in irrigant efficacy observed in this study are noteworthy. The chitosan nanoparticle-loaded irrigants were more effective against Staphylococcus aureus biofilms compared to Pseudomonas aeruginosa biofilms. This difference may be attributed to the structural and compositional variations between the biofilms formed by these species [23]. Pseudomonas aeruginosa biofilms are known to produce a more robust extracellular polymeric substance, particularly alginate, which may provide greater resistance to penetration and disruption by the nanoparticles [24]. Additionally, differences in cell wall structure between Gram-positive (Staphylococcus aureus) and Gram-negative (Pseudomonas aeruginosa) bacteria may influence their susceptibility to chitosan nanoparticles [25]. Gram-positive bacteria have a thick peptidoglycan layer but lack an outer membrane, potentially making them more susceptible to the disruptive effects of chitosan nanoparticles [26]. The superior performance of the 2% chitosan nanoparticle irrigant compared to 0.2% chlorhexidine is particularly significant, as chlorhexidine is widely regarded as the gold standard antimicrobial irrigant in many clinical applications [27]. Chlorhexidine acts by disrupting the cell membrane and causing precipitation of cytoplasmic contents, but its efficacy against biofilms is limited by poor penetration into the extracellular polymeric substance and inactivation by organic material [28]. In contrast, chitosan nanoparticles appear to overcome these limitations through their ability to penetrate the biofilm matrix and maintain their activity in the presence of organic material [29]. Furthermore, chitosan nanoparticles offer advantages in terms of biocompatibility and biodegradability compared to chlorhexidine, which has been associated with cytotoxicity and allergic reactions in some patients [30].

## Conclusion:

Chitosan nanoparticle-loaded irrigants, especially at higher concentrations, show superior biofilm disruption compared to conventional irrigants. Their unique ability to penetrate biofilms and effectively reduce bacterial viability highlights their promise as alternative agents for managing biofilm-associated infections. Further research is needed to validate their safety, stability and clinical applications.

## References

[1] Abidin T (2022). J Conserv Dent..

[2] Guerreiro-Tanomaru JM (2015). Braz Dent J..

[3] Aydin SA (2020). J Contemp Dent Pract..

[4] Abu Hasna A (2020). F1000Res..

[5] Pladisai P (2016). J Endod..

[6] Betancourt P (2020). Photobiomodul Photomed Laser Surg..

[7] Rödig T (2018). Quintessence Int..

[8] Choudhury P (2024). J Conserv Dent Endod..

[9] Juric BI (2014). Photomed Laser Surg..

[10] Shi Y (2019). Front Cell Infect Microbiol..

[11] Bhardwaj A (2013). Indian J Dent Res..

[12] Roshdy NN (2019). Acta Odontol Scand..

[13] Cherian B (2016). J Clin Diagn Res..

[14] Afkhami F (2021). Clin Oral Investig..

[15] Velardi JP (2022). J Endod..

[16] Álvarez-Sagües A (2021). Dent J (Basel)..

[17] Neuhaus KW (2016). J Endod..

[18] Bhuva B (2010). Int Endod J..

[19] Goel P (2022). Int J Clin Pediatr Dent..

[20] Nogueira L (2021). Eur Endod J..

[21] Mohmmed SA (2016). Dent Mater..

[22] Toljan I (2016). Acta Stomatol Croat..

[23] Mohmmed SA (2017). Microbiologyopen..

[24] Ibrahim RO (2021). J Contemp Dent Pract..

[25] Pacheco-Yanes J (2020). Clin Oral Investig..

[26] Balic M (2016). Lasers Med Sci..

[27] Munoz HR, Camacho-Cuadra K (2012). J Endod..

[28] Rodríguez-Figueroa C (2014). J Endod..

[29] Betancourt P (2019). Lasers Med Sci..

[30] de Almeida AP (2014). J Endod..

